# Review of electromyography onset detection methods for real-time control of robotic exoskeletons

**DOI:** 10.1186/s12984-023-01268-8

**Published:** 2023-10-24

**Authors:** Camila R. Carvalho, J. Marvin Fernández, Antonio J. del-Ama, Filipe Oliveira Barroso, Juan C. Moreno

**Affiliations:** 1 Neural Rehabilitation Group, Cajal Institute, Spanish National Research Council (CSIC), Madrid, Spain; 2 Electronic Technology Department, Rey Juan Carlos University, Madrid, Spain

**Keywords:** Electromyography, EMG onset, Movement detection, Real-time, Robotic exoskeletons

## Abstract

**Background:**

Electromyography (EMG) is a classical technique used to record electrical activity associated with muscle contraction and is widely applied in Biomechanics, Biomedical Engineering, Neuroscience and Rehabilitation Robotics. Determining muscle activation onset timing, which can be used to infer movement intention and trigger prostheses and robotic exoskeletons, is still a big challenge. The main goal of this paper was to perform a review of the state-of-the-art of EMG onset detection methods. Moreover, we compared the performance of the most commonly used methods on experimental EMG data.

**Methods:**

A total of 156 papers published until March 2022 were included in the review. The papers were analyzed in terms of application domain, pre-processing method and EMG onset detection method. The three most commonly used methods [Single (ST), Double (DT) and Adaptive Threshold (AT)] were applied offline on experimental intramuscular and surface EMG signals obtained during contractions of ankle and knee joint muscles.

**Results:**

Threshold-based methods are still the most commonly used to detect EMG onset. Compared to ST and AT, DT required more processing time and, therefore, increased onset timing detection, when applied on experimental data. The accuracy of these three methods was high (maximum error detection rate of 7.3%), demonstrating their ability to automatically detect the onset of muscle activity. Recently, other studies have tested different methods (especially Machine Learning based) to determine muscle activation onset offline, reporting promising results.

**Conclusions:**

This study organized and classified the existing EMG onset detection methods to create consensus towards a possible standardized method for EMG onset detection, which would also allow more reproducibility across studies. The three most commonly used methods (ST, DT and AT) proved to be accurate, while ST and AT were faster in terms of EMG onset detection time, especially when applied on intramuscular EMG data. These are important features towards movement intention identification, especially in real-time applications. Machine Learning methods have received increased attention as an alternative to detect muscle activation onset. However, although several methods have shown their capability offline, more research is required to address their full potential towards real-time applications, namely to infer movement intention.

## Background

Electromyography (EMG) has been used as an interface tool for human-robot interaction and rehabilitation systems [1]. In fact, EMG is a relevant biological signal to inform on the motion onset of the user and can be applied in different applications such as the control of robotic devices in rehabilitation, kinesiology, biomechanics and motor control during several movements of the upper and lower extremities [2–7].

Muscle activation can be defined as the degree to which a muscle is excited, encompassing both the number of activated muscle fibers and the rate of their discharge [8]. Therefore, muscle activation onset, which is commonly estimated from EMG, is a physiological variable related to the beginning of contraction of a given muscle. As there is a latency between the onset time and the final movement that involves action on tendons and bones, there is a time window after detecting movement intention that allows actuation and control of wearable robots such as an exoskeleton. Therefore, accurate and fast detection of muscle onset time can potentially be used to identify movement intention [2, 9] and assist the user in real time.

The first method applied to detect muscle onset was the offline visual inspection by a trained user [10]. Although it is a subjective approach, visual inspection can be considered to provide accurate EMG onset detection values [10, 11]. On the other hand, visual inspection lacks reproducibility and can hardly be used in real-time applications [10]. In this context, computerized methods started to be developed and applied, and are currently the main solution to detect EMG onset in robotics and neurofeedback fields.

Due to the stochastic nature of the EMG signal, detecting onset of muscle activation is a challenging task, especially when EMG signals are weak [12]. Furthermore, despite the extensive literature devoted to detection of muscle contraction episodes, there is not a gold standard approach yet [13] and there is a degree of disparity across studies with regard to the definitions and parameters applied in each algorithm. This leads to similar EMG onset detection methods, with different nomenclatures, making it difficult to identify the most appropriated method for a specific application [10, 11, 13].

Given the lack of agreement on a standardized method for EMG onset detection and its importance towards intuitive and natural EMG-based control systems, it is timely to explore methods for automatic EMG onset detection in this review and to compare the performance of some of the most commonly used ones towards online applications. Therefore, the main goal of this study was to review the state-of-the-art on EMG onset detection algorithms. This can boost the development of novel algorithms and finally create consensus towards a possible standardized method for EMG onset detection, which would also allow more reproducibility across studies. In fact, a recent international initiative called ’Consensus for experimental design in electromyography’ (CEDE project), which aims to guide decision-making in recording, analysis, and interpretation of EMG data has been carried out. Results of our study can feed the CEDE project, as this initiate encompasses definitions for terms used in the EMG literature, basic principles for recording and analyzing EMG and electrode selection [8, 14].

The second goal of our study was to evaluate the performance of the most commonly used onset detection methods [three threshold-based algorithms—single (ST), double (DT) and adaptive threshold (AT)] to determine muscle onset on experimental EMG data. This allowed us to to evaluate the potential of these methods towards the real-time control of wearable robots (e.g., robotic exoskeletons).

## Literature review

This review was based on the papers retrieved from the Scopus database using the following query strings:

TITLE-ABS-KEY ((emg OR electromyograph*) AND (onset AND detection))

and

TITLE((emg OR electromyograph*) AND (onset OR muscle OR movement) AND (detection OR activation)).

The first search returned a total of 245 papers and the second search 171, for a total of 416 possible publications. This research considered papers published until March 2022.

We applied the following exclusion criteria for our review:

- papers aiming at detecting muscle fatigue;
- use of additional sensors (e.g., inertial measurement units).

A total of 156 full-text journal articles were selected for analysis. The papers were analysed in terms of their application domain, pre-processing method and EMG onset detection method.

In the application domain, papers were classified as follows: Robotics, Clinical, Research, and Others. Specifically, papers that used EMG onset detection in the robotics domain (e.g., to control a robotic device) were classified as Robotics. Papers that presented results on the application of EMG onset techniques for clinical purposes or in the clinical setting were defined as Clinical. Research papers were those that proposed and/or tested a new technique of EMG onset detection. The remaining papers were classified as Others.

Pre-processing methods used to improve the quality of EMG before the application of the onset method itself were also analyzed in detail. Each EMG onset detection method was included in one of the following categories: Visual Inspection, Threshold-based, Statistical, Machine Learning or Others.

Regarding the EMG source type, from the total of 156 full-text journal articles selected for the analysis, 15 of them used intramuscular electromyography (iEMG) signals as the EMG source type where to test the performance of their algorithms at, whereas 145 articles used surface electromyography (sEMG) as the main EMG source type. This means that 4 articles tested onset detection algorithms in both sEMG and iEMG.

## Literature review results

**Fig. 1:**
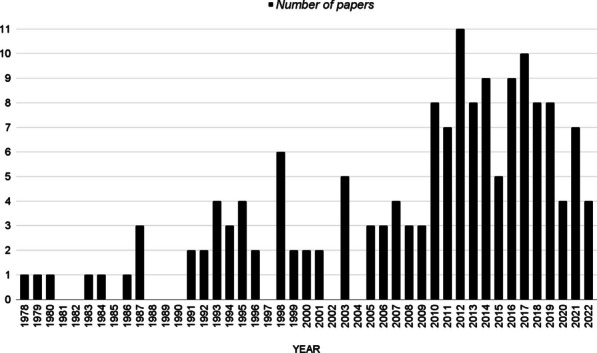
Number of publications on EMG onset detection methods per year reviewed in this study (period: 1978–2022; total articles: 156)

Figure 1 presents the number of publications on EMG onset detection methods along the years. After analyzing all papers selected, the pre-processing and EMG onset detection categories were defined according to the methods used and their relevance in terms of papers in the literature that applied each of them.

### Pre-processing methods

Pre-processing methods, used to improve the quality of EMG signals towards the extraction of meaningful information, usually add more computational time, which means a delay in real-time implementations. The pre-processing methods evaluated in this review were classified in the following categories: EMG Envelope, Teager-Kaiser Energy Operator (TKEO), Wavelet Transform and Others, which included those that did not fit in none of these categories. Different pre-processing methods were applied 90 times in the papers reviewed. Calculating the EMG envelope was the method most frequently used, followed by the TKEO method.

#### EMG envelope

**Fig. 2:**
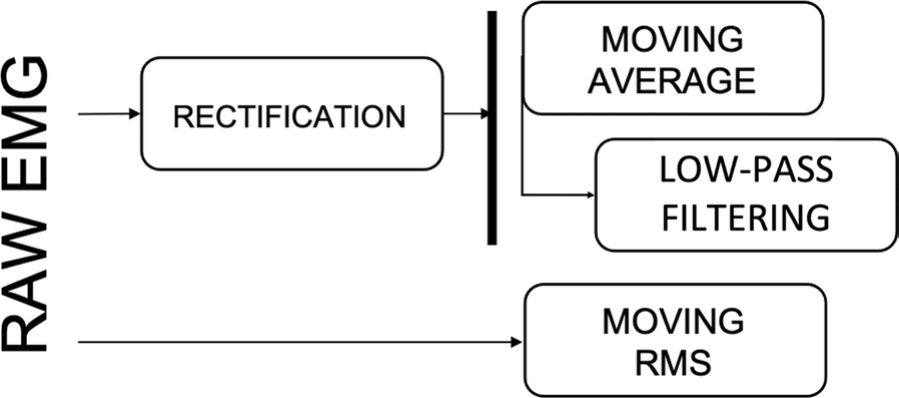
Comparison of the most common approaches to obtain the EMG envelope from the raw EMG signal

According to the CEDE project, EMG envelope is a smooth curve that tracks changes in the amplitude of an EMG signal over time [8]. Calculating the EMG envelope is a pre-processing method that can be obtained in several ways, as shown in Fig. 2.

To obtain the EMG envelope from raw signals, two main options are available: (1) low-pass filtering of the rectified signal; (2) root-mean-square (RMS) on raw EMG signal.

*Low-pass filtering of the rectified signal* One of the most common approaches to calculate the EMG envelope is to use a discrete version of traditional low-pass filters such as Butterworth or Chebyshev on the rectified EMG signal (obtained by computing the absolute value of the raw signal).. These filters can be considered as Infinite Impulse Response filters [15]. This approach was applied in: [16–26], with the Butterworth being the most predominant filter used.

*Moving average (MA)* According to the CEDE project, MA is defined as a method to smooth EMG data, that acts as a low-pass filter, reducing random fluctuations in the rectified or squared EMG signal [8].

This method was first used in the context of EMG onset detection by Maple-Horvart and Gilbey in 1992 [27]. After that, MA was applied to calculate EMG envelopes for EMG onset detection in several other papers: [13, 28–41].

The MA is calculated with a series of averages from successive segments, with or without overlapping windows. The consequence of its use is the attenuation of rapid variations through local averaging, but retention of slow variations [28], smoothing the signal and acquiring its envelope.

*Root-mean-square (RMS) on raw EMG signal* This approach ([28, 33, 42–51]) computes the RMS value of the signal within a window that “moves” across the raw EMG signal.

The RMS value measures the square root of the signal’s power. Therefore, it has a physical meaning. RMS is useful in many other applications [42]. EMG envelopes can be calculated from the RMS according to Eq. 1.

1 $$\begin{aligned} \begin{aligned} X_{RMS}=\sqrt{\frac{1}{N}\sum _{i=1}^{N}x_i^{2}} \end{aligned} \end{aligned}$$

where $x_{i}$ is the EMG value in the $i^{th}$ sample and *N* is the number of samples.

#### Teager–Kaiser energy operator (TKEO)

The TKE operator method ([17, 25, 38–40, 48, 52, 53, 53–67]) was first proposed by Teager in 1982 [68–70]. The results obtained in these studies suggested that the production of speech involved nonlinear processes. As a result, Teager derived the TKE operator in the discrete-time domain to compute the energy of a sound. This method has been extended to cover other continuous signals such as EMG [53].

The discrete TKE operator $\psi$ is defined in the time domain as:

2 $$\begin{aligned} \begin{aligned} \psi _{d}[x(n)]=x^{2}(n)-x(n+1)x(n-1) \end{aligned} \end{aligned}$$

where *n* is the sequence index and *x* the raw EMG signal. Considering a signal defined by Eq. 3:

3 $$\begin{aligned} \begin{aligned} x(n)=A cos[\omega _{0}(n)+\theta ] \end{aligned} \end{aligned}$$

where *A* is the amplitude, $\omega _{0}(n)$ is the angular frequency, and $\theta$ is the initial phase, the energy operator can be rewritten as defined in Eq. 4:

4 $$\begin{aligned} \begin{aligned} \psi _{d}[x(n)]\approx A^{2}sin^{2}(\omega _{0}) \end{aligned} \end{aligned}$$

Equation 4 shows that the TKEO is proportional to the instantaneous amplitude (*A*) and frequency ($\omega _{0}$) of the input signal. Therefore, TKEO is usually applied on EMG signals to extract motor unit activity by making the action potential spikes sharper and narrower, enhancing the muscle activation points [53].

Several studies have demonstrated that pre-processing using TKEO can improve the EMG onset detection with respect to different pre-processing methods [17, 52, 53, 57, 71].

#### Wavelet transform (WT)

Pre-processing of raw EMG using the wavelet transform was applied in the following papers: [32, 48, 52, 59, 62, 64, 67, 72–78].

The WT is one of many time-frequency representations used in signal processing. These transforms deconstruct a time domain signal into a sum of signals of different scales and time shifts, to produce a time-frequency representation of a time domain signal. WT is an effective tool to extract useful information from the EMG signal.[79].

#### Other pre-processing methods

The other pre-processing methods found in the literature were the Hilbert filter [9, 80–82], the Kalman filter [83], the Morphological Close Operator [38, 55], the Morphological Open Operator [38], the Multi Objective Optimization Genetic Algorithm [84], the Adaptative Linear Energy Detector [85], the use of an statistical criterion based on the amplitude distribution of EMG signal [86], the Constant False Alarm Rate method [87] and the Empirical Mode Decomposition [82].

### EMG onset detection methods

**Fig. 3:**
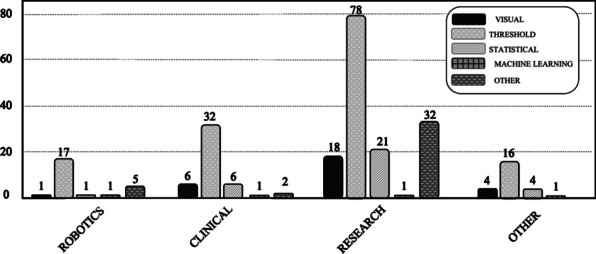
Number of publications included in each of the different EMG onset detection categories (Visual inspection—black; Threshold—light gray; Statistical—dashed grey; Machine Learning—bold gray squares; and Others—dashed bold gray) within each different application domain. Application domains considered were Robotics, Clinical, Research and Others

EMG onset detection methods are those that, when applied to the EMG signal (raw or pre-processed signal), allow the identification of the beginning of muscle activation. In the pasts, the onset of muscle activation could be detected using mainly the following methods: Visual inspection, Threshold-based and Statistical. Recently, other studies have tested different methods (especially Machine Learning based) to determine muscle activation onset, reporting promising results. In our study, all EMG onset detection methods that do not fit into none of the previously mentioned categories were classified as ”Other EMG onset Detection Methods”. Figure 3 shows the number of papers that applied each of these categories within each different application domain (Robotics, Clinical, Research and Others). EMG onset detection has been applied in the application domain ’Research’ more than in all the other domains together.

#### Visual inspection

Visual inspection entails subjectivity and needs to be performed by an expert. There are no criteria established on how to carry out the visual inspection technique, although it is usually employed to detect the earliest rise in EMG activity above the steady-state (i.e., basal activity) [50, 88–94].

Despite being a subjective technique, visual inspection can be used to validate automatic EMG onset detection methods, serving as a gold-standard to develop computerized EMG onset detection methods. The visual detection of EMG onset has been widely referred in the literature: [11, 17, 21, 24–26, 32, 33, 36, 43, 49, 52, 57, 60, 60, 73, 95–102].

#### Threshold-based methods

The label ’THRESHOLD’ in Fig. 3 encompasses the use of one or more threshold-based methods, which are thoroughly described in this section, in each of the papers analyzed in this review. Threshold-based are the most common EMG onset detection methods found in the literature, being tested 253 times across the 156 papers analyzed (i.e., several papers tested and/or compared more than one different method based on EMG threshold).

In this approach, one attributes a threshold to discriminate between baseline activity and muscle activation. Thresholding is widely used due to its simplicity, speed and reliability. The simplicity of thresholding lies in its straightforward implementation. Additionally, thresholding is computationally efficient, making it suitable for real-time analysis of EMG signals and handling large datasets. Regarding its reliability, thresholding is a robust method that has been used in numerous studies in the literature. Although thresholding may not always provide the most accurate detection of EMG onset, it remains a popular choice in EMG signal processing. Nonetheless, there is lack of agreement among researchers on a standardized threshold method for EMG onset detection [46].

Cavanagh et al. were the first to propose the use of a threshold-based method [103]: authors investigated the dependence of electromechanical delay in the human elbow flexor group upon selected initial conditions at the time of muscle activation. Most common strategies followed to set threshold values are based on the baseline amplitude characteristics of the EMG signal, such as the mean or standard deviation. Some researchers name this strategy as the Shewhart protocol [16, 30, 104].

Some of the signal characteristics that can be considered to select the threshold are the following:

- Standard Deviation (SD);
- Period of time;
- % Maximum Voluntary Contraction;
- % Maximum EMG Amplitude.

Threshold-based methods can be classified in three different categories: Single Threshold (ST), Double Threshold (DT) and Adaptive Threshold (AT).

*Single threshold (ST)* ST method is the most predominant EMG onset detection method found in the literature: [2, 11, 13, 16–19, 22–24, 27, 29–32, 35–41, 43, 45–48, 51–53, 55–57, 59, 60, 63, 66, 67, 74, 77, 81–84, 86, 95, 96, 99, 103, 105–133].

ST compares the amplitude of the EMG signal (raw or EMG envelope) with a previously selected threshold. The onset is detected when the EMG amplitude is bigger than the threshold.

This method can be considered the most intuitive and standard computer-based method of time-locating the onset of muscle contraction activity [2].

ST can be useful to overcome some of the problems related to visual inspection. However, results of applying ST strongly depend on the choice of the threshold [134], which can lead to false positives in noisy signals. In theses cases, it is advisable to work on the EMG envelope, which smooths the signal and improves the onset detection.

*Double threshold (DT):* The DT method was applied in the following studies: [2, 10, 19–21, 24, 33, 34, 42, 51, 55, 65, 73, 82, 83, 87, 94, 95, 100, 115, 119–121, 123, 127, 128, 135–151].

To overcome some of the problems associated with ST, Lidierth et al. introduced the DT method in 1986 [135]. This method adds a second threshold to determine the muscle activation onset time, with the final goal of avoiding false positives and enhance EMG onset detection precision. A common strategy when applying DT method is to define an amplitude threshold, similar to what is done in ST. If the signal amplitude is higher than this threshold for a certain amount of time or samples (second threshold), then muscle activation is detected with DT. Due to the stochastic characteristics of the EMG signal, it is normally necessary to use a pre-processing method to obtain the signal envelope and then apply the second threshold.

*Adaptive threshold (AT)* The AT method can be applied directly on the raw EMG signal. AT segments the signal using the signal-to-noise (SNR) [61] or the energy value [85] to adapt the threshold of muscle activation by windows. As the SNR is the relative power of wanted EMG to unwanted signal components that are contained in the overall signal [8], this threshold method can be considered as an improvement of ST method, as it adapts its threshold value according to the EMG window being analyzed, which might enable a more precise EMG onset detection over time. AT was applied in the following works: [11, 52, 54, 55, 85, 97, 118, 121, 152–156].

### Statistical methods

The onset of muscle activation can be detected by evaluating the statistical properties of the EMG signal before and after a possible change in model parameters [115]. Two main statistical approaches can be identified in the literature: the Approximated Generalized Likelihood Ratio (AGLR) and the Cumulative Sum (CUSUM).

*Approximated generalized likelihood ratio (AGLR):* The AGLR method was applied in the following publications: [10, 17, 37, 52, 57, 58, 67, 72, 77, 83, 93, 94, 100, 115, 121, 128, 157–160].

Sometimes also referred to as “Maximum Likelihood Estimator”, this method was first proposed as a change detection algorithm, with its first use in the context of muscle activity detection being presented in Hogan et al., in 1980 [157]. In short, the AGLR algorithm calculates an estimate of muscle activity as a function of the mean and variance of the activity level [121].

By using a log-likehood ratio test *g*(*k*) [66], the AGLR method detects if there is muscle contraction or not.

The log-likelihood ratio test is defined by the following equation:

5 $$\begin{aligned} \begin{aligned} g(k)=ln\left( \prod _{k=1}^{r} \frac{p1(Y_{n}|H_{1})}{p0(Y_{n}|H_{0})}\right) \end{aligned} \end{aligned}$$

where *ln* represents the natural logarithm, *Y*(*n*) represents the series of EMG samples, *k* the index of the product, *r* is the total length of the series, *p*1 and *p*0 represent the probability density function corresponding to the alternative hypothesis $H_{1}$ (i.e., there are changes in the statistical properties of the EMG sequence) and the null hypothesis $H_{0}$ (i.e., there are no changes in the statistical properties of the EMG sequence), respectively.

If the log-likelihood *g*(*k*) value is smaller than a pre-defined threshold, it indicates that the muscle is relaxed, whereas EMG onset is detected if *g*(*k*) value exceeds the threshold.

*Cumulative sum (CUSUM):* This method was used in: [67, 72, 95, 109]. CUSUM was first proposed by Ellaway in 1978 [161] with applications on the analysis of histograms.

The first study to propose the use of CUSUM to detect EMG onset was Chanaud et al., in 1991 [109], which used this method to determine how the different regions of the biceps femoris activated in a cat during a broad range of limb movements.

The CUSUM method works as follows [161]: a reference level (k), dependent on the task to be performed and selected in a previous training phase, is subtracted from each of the series of points on the signal (*x*1, *x*2, ..., *xi*, ..., *Xn*). The result of these subtractions, shown in Eq. 6, is a new series of points (Si) which are formed by adding up these differences consecutively.

6 $$\begin{aligned}S1 &= (x1-k) \\S2 &= (x1-k)+(x2-k) \end{aligned}$$

The CUSUM chart is defined as the sequential plot of the values of Si, expressed by the Eq. 7:

7 $$\begin{aligned} \begin{aligned} Si = \sum (xi-k) \end{aligned} \end{aligned}$$

The CUSUM technique has a smoothing action on the data [161] and the EMG onset detection is determined by a previous threshold, which can be defined by a training phase (see [72] for more details).

*Other statistical methods* Other statistical methods were also used in the following papers: [25, 49, 50, 60, 61, 75, 145, 147, 160, 162, 163].

#### Machine learning methods

Machine learning (ML), which is a discipline within the field of Artificial Intelligence, has recently gained increasing popularity due to the ability to extract patterns and information from complex and high-dimensional datasets. In the context of EMG onset detection, ML algorithms can automatically learn and adapt to the characteristics of the EMG signals, enabling the development of highly accurate and efficient detection methods. Machine learning-based algorithms were found in [33, 65, 164, 165].

Di Nardo et al. [165] evaluated a novel machine-learning-based approach (DEMANN) for detecting muscle activation onset/offset timing from sEMG signals. The study trained a neural network and evaluated DEMANN’s performance on simulated and real sEMG signals. DEMANN was validated against different reference algorithms, including the DT method. The study found that DEMANN provided a reliable prediction of muscle onset/offset and was minimally affected by SNR variability.

Trigili et al.[164] presented a ML-based algorithm able to detect users’ motion intention based on EMG signals and assessed its applicability towards the control of an upper-limb exoskeleton for people with severe arm disabilities. The algorithm was able to detect the onset of muscle activation before the actual movement, and its computational load was compatible with real-time applications. The study concluded that the proposed algorithm was promising for controlling upper-limb exoskeletons in real-time applications, and for assisting people with severe arm disabilities in performing functional tasks.

Dow et al. [33] presented the development of an algorithm to detect inspiratory events from EMG signals. A state-machine was utilized for classification and inspirations were detected with  98% accuracy in anesthetized and awake rats. The proposed algorithm can be explored in humans, as it may be useful for individuals requiring assisted ventilation.

Ghislieri et al. [65] introduced and validated a new approach to detect muscle activation intervals from sEMG signals using long short-term memory (LSTM) recurrent neural networks. The performance of the proposed LSTM-based muscle activity detector was compared with two other widely used approaches: TKEO and DT method. The study included simulated and real sEMG signals from healthy individuals, orthopedic patients, and neurological patients. Results showed that the LSTM outperformed the other approaches. The proposed algorithm overcomes the main limitations of other tested approaches and works directly on sEMG signals without the need for background-noise and SNR estimation.

Despite the growing interest in using ML techniques for EMG onset detection, there is currently no consensus on a reference method. As noted by Di Nardo et al. [165], a standardized approach for evaluating and comparing the performance of ML algorithms for EMG onset detection is still lacking. While several studies have proposed different ML approaches and achieved promising results, the absence of a reference method makes it challenging to classify these methods.

#### Other EMG onset detection methods

Other methods can be classified as: Energy-based methods [34, 85, 92], Entropy-based methods [12, 25, 38, 125, 146], Mathematical/numerical techniques [9, 16, 55, 166, 167], Computer Vision [127], Slope / discontinuities detectors [51, 62, 76] and External stimulation [168, 169].

## Experimental protocol

The second goal of this study was to compare the performance of the three most commonly used methods for EMG onset detection, having in mind their potential towards online control of robotic exoskeletons for gait assistance or rehabilitation. As shown in Fig. 3, Threshold-based methods are the most commonly used in all application domains (Robotics, Clinical, Research and Others). For this reason, ST, DT and AT were tested on real data (iEMG and sEMG signals) obtained during motor tasks involving knee and ankle joints. This was done through simple tasks that could be used as a paradigm for comparison (such as knee and ankle flexion-extension), rather than gait, to minimize the influence of possible external factors (e.g., mechanical artifacts) on the evaluation of these methods. Furthermore, knee and ankle flexion-extension are important tasks during gait [170–173].

### Participants

Three healthy subjects participated in this study. All procedures were approved by a local Ethics Committee (“Ethics Committee of Clinical Research with Medicines of the Hospital Complex of Toledo”), as well as by the Spanish Agency of Medicines and Medical Devices (AEMPS)—record 721/19/EC. All subjects volunteered to participate in the study, were informed about the procedures and possible adverse effects, and signed the informed consent to participate.

### Data collection

EMG data were recorded while participants performed ankle dorsiflexion-plantarflexion and knee flexion-extension movements, using an EMG amplifier (Quattrocento, OT Bioelettronica, Torino, Italy) and a sampling frequency of 10,240 Hz. sEMG and iEMG were recorded from Tibialis Anterior (TA) and Vastus Lateralis (VL). For sEMG recordings, bipolar electrodes (Ag-AgCl, Ambu Neuroline 720, Ambu, Ballerup, Denmark) were used. For iEMG recordings, intramuscular thin wire electrodes (Fi-Wi2, Spes Medica, Genova, Italy) were used. More information on the protocol can be found in [174].

The selected number of muscles was kept to a minimum to simplify the procedure due to the invasiveness of the iEMG electrodes. On the other hand, testing the feasibility and performance of different threshold-based controllers can be carried out with such reduced number of EMG signals, as demonstrated in our previous study, where EMG-based controllers were used online to trigger the beginning of walking steps while healthy volunteers walked with an exoskeleton [175]. And, finally, these two muscles have an important role during gait [174].

### Data processing

EMG onset was automatically detected offline using each of the three threshold-based methods (ST, DT and AT) on the EMG data recorded from each muscle (TA and VL) and participant, during knee and ankle flexion-extension trials. Threshold values were determined individually for each subject and task, and were based on the standard deviation (SD) of the baseline activity of each EMG recording through visual inspection.

Threshold values were defined between 1 and 3 times the SD of the EMG baseline, according to a training phase performed with each subject (see Table 1).

**Table 1:** Values of EMG onset threshold used for ankle and knee tasks, for the three methods (ST, DT, AT)

Method	Task	S01	S02	S03
ST	Ankle	2 SD	2 SD	1 SD
	Knee	2 SD	1.5 SD	2 SD
DT	Ankle	1 SD	2.5 SD	1 SD
	Knee	3 SD	2.5 SD	3 SD
AT	Ankle	2 SD	2 SD	1.5 SD
	Knee	2 SD	2.5 SD	1 SD

The threshold values were based on the standard deviation (SD) of the baseline activity presented by each subject, for the two different tasks. S01 - Subject 1. S02 - Subject 2. S03 - Subject 3

The DT method has to be performed on the EMG envelope due to the variation of the EMG amplitude over time (the second threshold). EMG envelopes were calculated using a Butterworth low-pass filter of second-order with a cut-off filter of 6 Hz [15]. The time required to ascertain muscle activation onset with DT method was 2.50 ms, i.e., EMG envelopes needed to stay above the first threshold for at least 2.5 ms in order for muscle activation to be detected offline.

The windows used in AT had a duration of 1/N of the total recording, where N is the number of movement cycles performed by the subject.

To analyze the performance of each method, the number of false/positive EMG onset detection events, the detection time and the processing time of each method were compared. The analysis of false positives/negatives was visually performed offline: any event labeled as the onset of EMG activation by a given method was considered as a false positive when an expert did not perceived it as a true onset of muscle activation; on the other hand, false negatives were those events labeled by an expert (offline, when assessing data visually) as an onset of muscle activation, but these were not detected by a given method. Furthermore, the difference between the onset timing calculated by each threshold-based method and the visual inspection (which was used as reference) was calculated for each cycle, task and subject. The mean difference was calculated for each method and subject. For this step, the first and last cycle of movement in both knee and ankle task performance were not considered, to exclude possible transients in the signal. Processing times required by each method were also calculated.

### Results

The mean processing times needed by the computer (2,7 GHz Intel Core i7 processor) to compute the three threshold-based methods (ST, DT and AT), for each cycle of movement, are shown in Table 2.

**Table 2:** Mean ± SD processing time spent per cycle and its standard deviation, for each EMG onset detection method: ST, DT and AT

Method	Processing time/ cycle [ms]
ST	1.250 ± 0.23
DT	14.302 ± 4.65
AT	1.905 ± 0.14

DT method required more processing time (almost 10 times more) than the other two methods. The lengthy process required by DT to calculate EMG oset is due to the use of a pre-processing method (EMG envelope).

ST and DT methods obtained one false positive in a total of 124 cycles, which corresponds to 0.8% of detection error. AT obtained 9 false positives, which corresponds to a 7.3% of error detection. Table 3 represents the performance of the different threshold-based algorithms. This was done by assessing the mean time differences that each automatic method needed to detect muscle activation onset from both sEMG and iEMG signals, compared to the visual inspection.

**Table 3:** Comparison of the mean ± SD detection times between ST, DT and AT methods with respect to visual inspection, in both sEMG and iEMG recordings

Method	Subject 01 [ms]	Subject 02 [ms]	Subject 03 [ms]
ST	245.92 ± 116.81	332.10 ± 249.13	424.42 ± 261.03
DT	210.31 ± 180.10	502.47 ± 245.90	574.57 ± 192.89
AT	233.08 ± 132.52	336.67 ± 306.40	347.63 ± 63.21

All values are positive, which means that automatic detection was always done with a delay with respect to the visual inspection

**Fig. 4:**
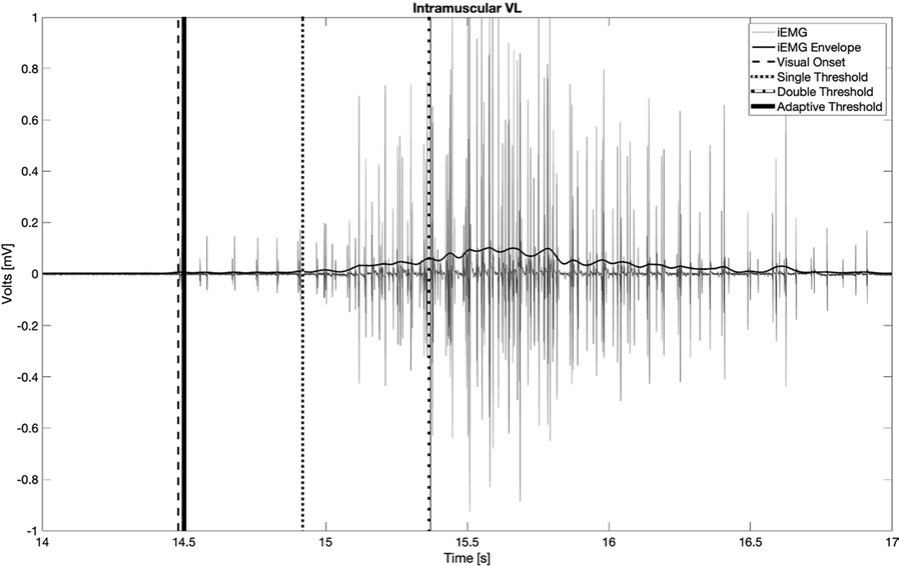
Individual example of EMG onset detection using four different methods (visual inspection (dashed line), ST (dotted line), DT (spaced dotted line) and AT (bold)) on iEMG recordings from Tibialis Anterior (TA) during ankle flexion-extension. Methods were applied offline. ST—Single Threshold. DT—Double Threshold. AT—Adaptive Threshold

Considering the global performance of each threshold-based method, higher time precision was achieved using AT, which detected the onset of muscle activation, on average, 0.30 s after the visual inspection. ST and DT detected activation onset, on average, 0.33 s and 0.43 s after the visual inspection, respectively. Figure 4 shows an example of the detection timing for each of the three threshold-based methods assessed, which were applied on iEMG data from TA during one cycle of ankle dorsiflexion-plantarflexion task. Although the iEMG presented in Fig. 4 is very selective and presents clear individual action potentials, the iEMG signal was not always detected at the level of individual motor units, making a signal decomposition into individual motor unit action potentials not a viable option.

Table 4 presents differences in terms of detection times when applying each of the threshold-based methods on sEMG and iEMG recordings. Muscle activation onset was detected before in iEMG signals for all subjects, using the three threshold-based methods, with the exception of DT in subject 02. On average, ST, DT and AT methods detected muscle activation 0.17, 0.02 and 0.16 s before in iEMG than in sEMG recordings, respectively.

**Table 4:** Comparison of the mean ± SD EMG onset detection times between iEMG and sEMG for ST, DT and AT methods

Method	Subject 01 [ms]	Subject 02 [ms]	Subject 03 [ms]
ST	115.15 ± 297.19	233.67 ± 152.21	163.11 ± 261.77
DT	46.86 ± 524.71	− 7.35 ± 68.01	26.93 ± 83.99
AT	157.92 ± 364.06	293.94 ± 323.81	21.31 ± 297.51

Positive values indicate that muscle activation onset was detected earlier in iEMG signals than in sEMG signals (for the same muscle and task), while negative values indicate the opposite, i.e., muscle activation onset was detected earlier in sEMG signals than in iEMG signals (also for the same muscle and task)

## Discussion

EMG has been increasingly applied in the research field as part of intuitive and natural control in human-robot interaction and rehabilitation systems for restoration of human movement and control of external devices from recordings of neural activity. One key component of these systems is the detection of movement intention (*i.e*., the onset of muscle activity). Given the lack of agreement on a standardized method for EMG onset detection and its importance towards intuitive and natural EMG-based control systems, it is timely to explore methods for automatic EMG onset detection. The first goal of our study was to review the state-of-the-art on EMG onset detection algorithms.

A total of 156 papers published until March 2022 were included in the review. The papers were analyzed in terms of application domain, pre-processing method and EMG onset detection method.

Regarding the application domain, EMG onset detection has been applied in the ’Research’ field more than in all the other domains together (i.e., Robotics, Clinical and others). This highlights the need of a standardized method for EMG onset detection, which may boost the translation from bench to bedside so that the onset of muscle activity could be used in more Clinical and Robotics applications, for example.

A total of 40% of all papers reviewed used a pre-processing technique before applying an onset detection method. Fast computational pre-processing methods are important to improve the quality of the EMG signal towards the extraction of information related with movement intention. EMG envelope was the most frequently applied pre-processing method across the reviewed papers. Although other authors have considered the EMG envelope and the TKEO as EMG onset detection methods [60], we have classified them as pre-processing methods, as previously done by others [53].

Threshold-based methods were found to be the most commonly used methods for EMG onset detection, for all application domains. Thresholding is a widely used method for detecting EMG onset due to its simplicity, speed and reliability. Recently, other studies have tested different methods (especially Machine Learning - ML - based) as an alternative to detect muscle activation onset, reporting promising results. One of the main advantages of ML-based methods is their ability to learn patterns from a dataset without the need for explicit feature extraction, which can otherwise be difficult and time-consuming. This can lead to more robust and generalizable models that can be applied to a wider range of datasets. Additionally, ML algorithms can adapt to changes in the data over time, allowing for the development of more adaptive and personalized systems. There are, on the other hand, some limitations of ML-based methods. One potential concern is the risk of overfitting, where the algorithm may learn the specific characteristics of the training data notably that it prevents it to generalize well to new data. This can, however, be mitigated through the use of appropriate validation methods and careful selection of training data. Additionally, ML-based methods can be computationally expensive and may require significant amounts of data to train. In this sense, more research is required to address the full potential of ML towards real-time applications, namely to infer movement intention.

We consider that our study fills a gap in the literature by providing a comprehensive classification framework for EMG onset detection methods. This framework not only enables a better understanding of the existing methods but also offers a practical tool for researchers and practitioners to easily navigate the field and select appropriate techniques for their specific requirements. This can also boost the development of novel algorithms and finally create consensus towards a possible standardized method, which would also allow more reproducibility across studies. Previously, 19 papers reviewed onset detection methods [10, 11, 19, 34, 36, 49, 53, 54, 57, 60, 71, 77, 83, 95, 96, 115, 119, 121, 128], although none of them has performed an extensive classification and definition of the available methods. From these 19 papers, only 3 of them had the review of onset detection methods as their main goal [10, 54, 115].

The second goal of our study was to evaluate the performance of the most commonly used onset detection methods (single (ST), double (DT) and adaptive threshold (AT)) to determine muscle onset on experimental EMG data (sEMG and iEMG). This allowed us to evaluate the potential of these methods towards the real-time control of wearable robots (e.g., robotic exoskeletons).

ST was the fastest of the three threshold-based methods to calculate EMG onset both in iEMG and sEMG data. This result can be explained by the low computational processing complexity required by ST, as well as the unnecessary employment of pre-processing methods, i.e., it can be applied on raw EMG data. On the other hand, ST is less precise than the other two threshold-based methods, as it uses a fixed threshold for the entire EMG signal. This can increase the number of false positives/negatives in terms of onset detection events. In that case, the use of TKEO as a pre-processing method can improve the accuracy of onset detection compared to ST alone [71]. Taking into account the advantages and disadvantages of ST, we consider that ST suits simple tasks (such as EMG-based static rehabilitation) best, where a fast response of the system is more important than its precision, and false negatives/positives do not mean security problems to the user.

DT was the slowest of the three methods to calculate EMG onset. This is due to the need of calculating EMG envelope (in addition to the second threshold), which adds more processing time and, thus, can compromise its use in real-time applications, especially when a fast detection is required. DT is more precise that ST to calculate EMG onset, as it uses two different thresholds (amplitude andd time) to detect the onset of the muscle activation. Therefore, it can be a robust solution to process EMG recordings offline [19].

Considering the global performance of each threshold-based method, higher time precision was achieved using AT, which detected the onset of muscle activation, on average, 0.30 s after the visual inspection. AT adapts the threshold of onset detection by segmenting the whole signal into small windows, allowing a more precise detection of muscle activation. AT can be a good option for real-time applications that need more precision and relatively fast processing times in terms of detection of muscle activation [153].

In summary, the choice of the most appropriate EMG onset detection method depends on the type of application and its requirements. A trade-off between processing speed, accuracy and time precision should be taken into account before defining which onset method best fits the application in question.

Muscle activation onset was detected before in iEMG signals for all subjects, using the three threshold-based methods, with the exception of DT in subject 02. Given that early detection of muscle contraction can be critical for real-time applications, iEMG signals should also be considered as an alternative to be used as input for human–machine interfaces [9], e.g., for the intuitive control of robotic exoskeletons, where timing is crucial for an efficient control strategy. Additionally, iEMG signals present higher SNR and less cross-talk, when compared with sEMG signals [174].

## Conclusions

EMG can be used to infer movement intention and trigger exoskeletons and prostheses. However, determining the onset of muscle activation from EMG activity is not a trivial task. The first conclusion of this paper is that there is still no agreement on a standardized method for EMG onset detection (neither offline nor online), which hinders reproducibility across studies. Therefore, this study organized and classified the existing EMG onset detection methods in an attempt to bring additional interest to the field and create consensus towards a possible standardized method for EMG onset detection, which would also allow more reproducibility across studies. Despite the lack of standardized methods, the research interest has been growing along the years, with a soaring number of publications in the field.

A total of 156 papers published until March 2022 were analyzed in terms of application domain (Robotics, Clinical, Research and Others), pre-processing method and EMG onset detection method. Pre-processing methods are used to improve EMG quality towards the extraction of meaningful information, although this adds more computational time and might be a drawback towards real-time applications. EMG envelope, which represents the average activation of the EMG signal, was found to be the most used pre-processing method before applying algorithms aiming at detecting onset of muscle activity.

Threshold-based methods were found to be the most commonly used methods for EMG onset detection. On the other hand, Machine Learning (ML) methods have recently received increased attention as an alternative to detect muscle activation onset, reporting promising results. However, more research is required to address the full potential of ML towards real-time applications, namely to infer movement intention.

This study also evaluated the performance of the most commonly used onset detection detection methods (single (ST), double (DT) and adaptive threshold (AT)) to determine muscle onset on experimental EMG data. This allowed us to to evaluate the potential of these methods towards the real-time control of wearable robots. Results showed that DT required more processing time, which led to increased average onset timing detection compared to the other two methods, while ST and AT were faster in terms of EMG onset detection time, especially when applied on iEMG data. These are very important features towards movement intention identification. In that sense, this opens the window to further explore these methods in real-time applications such as the intuitive control of exoskeletons. In any case, the choice of the most appropriate EMG onset detection method depends on the type of application and its requirements. A trade-off between processing speed, accuracy and time precision should be taken into account before defining which onset method best fits the application in question.

One limitation of this study is its reduced sample size (n = 3 healthy subjects), which affects the likelihood of obtaining statistical differences and, thus, the generalizability of the results. However, data collected served the purpose of achieving the secondary goal of this study, allowing us to assess the advantages and limitation of each of the most used methods for EMG onset detection.

## Data Availability

The datasets used and/or analyzed in this study are available from the corresponding author upon reasonable request

## Abbreviations

AGLR: Approximated generalized likehood ratio
AT: Adaptive threshold
CUSUM: Sumulative sum
DT: Double threshold
EMG: Electromyography
iEMG: Intramuscular electromyography
LSTM: Long short-term memory
MA: Moving average
ML: Machine learning
RMS: Root-mean-square
SD: Standard deviation
sEMG: Surface electromyography
SNR: Signal-to-noise
ST: Single threshold
TA: Tibialis anterior
TKEO: Teager–Kaiser energy operator
VL: Vastus lateralis
WT: Wavelet transform
